# Assessment of Changes in Rural and Urban Primary Care Workforce in the United States From 2009 to 2017

**DOI:** 10.1001/jamanetworkopen.2020.22914

**Published:** 2020-10-28

**Authors:** Donglan Zhang, Heejung Son, Ye Shen, Zhuo Chen, Janani Rajbhandari-Thapa, Yan Li, Heesun Eom, Daniel Bu, Lan Mu, Gang Li, José A. Pagán

**Affiliations:** 1 Department of Health Policy and Management, College of Public Health, University of Georgia, Athens; 2 Department of Epidemiology & Biostatistics, College of Public Health, University of Georgia, Athens; 3 Department of Population Health Science and Policy, Icahn School of Medicine at Mount Sinai, New York, New York; 4 Department of Obstetrics, Gynecology, and Reproductive Science, Icahn School of Medicine at Mount Sinai, New York, New York; 5 Department of Geography, University of Georgia, Athens; 6 School of Medicine and Health Management, Tongji Medical College, Huazhong University of Science and Technology, Wuhan, Hubei, China; 7 Department of Public Health Policy and Management, School of Global Public Health, New York University, New York

## Abstract

**Importance:**

Access to primary care clinicians, including primary care physicians and nonphysician clinicians (nurse practitioners and physician assistants) is necessary to improving population health. However, rural-urban trends in primary care access in the US are not well studied.

**Objective:**

To assess the rural-urban trends in the primary care workforce from 2009 to 2017 across all counties in the US.

**Design, Setting, and Participants:**

In this cross-sectional study of US counties, county rural-urban status was defined according to the national rural-urban classification scheme for counties used by the National Center for Health Statistics at the Centers for Disease Control and Prevention. Trends in the county-level distribution of primary care clinicians from 2009 to 2017 were examined. Data were analyzed from November 12, 2019, to February 10, 2020.

**Main Outcomes and Measures:**

Density of primary care clinicians measured as the number of primary care physicians, nurse practitioners, and physician assistants per 3500 population in each county. The average annual percentage change (APC) of the means of the density of primary care clinicians over time was calculated, and generalized estimating equations were used to adjust for county-level sociodemographic variables obtained from the American Community Survey.

**Results:**

The study included data from 3143 US counties (1167 [37%] urban and 1976 [63%] rural). The number of primary care clinicians per 3500 people increased significantly in rural counties (2009 median density: 2.04; interquartile range [IQR], 1.43-2.76; and 2017 median density: 2.29; IQR, 1.57-3.23; *P* < .001) and urban counties (2009 median density: 2.26; IQR. 1.52-3.23; and 2017 median density: 2.66; IQR, 1.72-4.02; *P* < .001). The APC of the mean density of primary care physicians in rural counties was 1.70% (95% CI, 0.84%-2.57%), nurse practitioners was 8.37% (95% CI, 7.11%-9.63%), and physician assistants was 5.14% (95% CI, 3.91%-6.37%); the APC of the mean density of primary care physicians in urban counties was 2.40% (95% CI, 1.19%-3.61%), nurse practitioners was 8.64% (95% CI, 7.72%-9.55%), and physician assistants was 6.42% (95% CI, 5.34%-7.50%). Results from the generalized estimating equations model showed that the density of primary care clinicians in urban counties increased faster than in rural counties (β = 0.04; 95% CI, 0.03 to 0.05; *P* < .001).

**Conclusions and Relevance:**

Although the density of primary care clinicians increased in both rural and urban counties during the 2009-2017 period, the increase was more pronounced in urban than in rural counties. Closing rural-urban gaps in access to primary care clinicians may require increasingly intensive efforts targeting rural areas.

## Key Points

**Question:**

What are the changes in the rural-urban distribution of the primary care workforce in the US from 2009 to 2017?

**Findings:**

In this cross-sectional study of 3143 US counties (1167 urban and 1976 rural) using county-level data, the density of primary care clinicians increased significantly in both rural and urban counties from 2009 to 2017. The increase in primary care clinician density was more pronounced in urban counties compared with rural counties.

**Meaning:**

In this study, the density of primary care clinicians increased overall, yet rural-urban disparities in the primary care workforce are increasing in the US.

## Teaser

This cross-sectional study examines and compares the primary care workforce and its growth between urban and rural counties or county-equivalents from 2009 to 2017 in the US.

## Introduction

In recent decades, disparities in population health between rural and urban residents have widened in the US.^1^ One notable disparity is the higher prevalence of coronary heart disease, stroke, and all-cause mortality among rural vs urban populations.^2,3,4,5^ Primary care workforce supply is a key factor in access to primary care clinicians,^6^ which is necessary for improving quality of care and population health.^7^ However, rural-urban disparities occur in access to primary care clinicians.^8^ Despite efforts to address these disparities, the shortages of rural primary care clinicians have persisted.^7,9,10^ Lack of an adequate number of rural physicians is a major barrier preventing individuals living in rural and underserved areas from accessing coordinated and integrated care that is essential to healthy and productive lives.^11^

In the US health care system, the main primary care workforce includes primary care physicians such as internists, family physicians, and general practitioners, as well as nonphysician clinicians such as nurse practitioners and physician assistants.^12^ Historically, people living in rural areas of the US have experienced limited access to primary care clinicians compared with those living in urban areas.^13,14^ More than one-third of rural US residents live in federally designated health professional shortage areas^15,16^ and approximately 82% of rural counties are classified as medically underserved regions.^17^ The shortage of primary care physicians in rural areas is associated with longer travel distance to accessing services, which may mitigate the prevention and management of the prevalent chronic diseases.^18,19^ While this shortage has been described and incentives to attract more physicians to rural areas have been adopted, few analyses have characterized the potential changes in urban-rural physician distributions over the past 10 years.^20^

This study examines and compares the primary care workforce and its growth between urban and rural counties or county-equivalents from 2009 to 2017 in the US. Rural clinician shortages may be associated with the widening gap in population health outcomes between rural and urban residents.

## Methods

Data on all registered primary care clinicians who had an active National Provider Identifier record in the National Plan and Provider Enumeration System from 2009 to 2017 were obtained from the Centers for Medicare & Medicaid Services.^21^ Data were analyzed from November 12, 2019, to February 10, 2020. We used the National Uniform Claim Committee Health Care Provider Taxonomy code in the National Plan and Provider Enumeration System to identify the main primary care workforce. The workforce included general practitioners, physicians who practiced family medicine and those who specialized in internal medicine only (without other subspecialty), nurse practitioners, and physician assistants.^12^ All the data on health care clinicians were deidentified and linked with the American Community Survey database collected from the US Census Bureau.^22^ Per Common Rule, this study was exempt from institutional review board review because all data were deidentified and publicly available and, therefore, did not qualify as human subjects research. We followed the Strengthening the Reporting of Observational Studies in Epidemiology (STROBE) reporting guideline for cross-sectional studies to report our findings.^23^

Primary outcome measures included the density of primary care physicians, nurse practitioners, and physician assistants in each county derived from the number of each type of primary care clinician per 3500 residents in each county. This is the definition used by the Health Resources and Services Administration to identify primary care health professional shortage areas.^24^ According to the Urban-Rural Classification Scheme for Counties defined by the Centers for Disease Control and Prevention National Center for Health Statistics in 2013,^15^ we classified counties as rural or urban, assuming that their rural or urban status did not change during the study period.^15^ Counties with an urban-rural score of 1 to 4 were classified as metropolitan (urban [≥250 000 inhabitants]) and those with a score of 5 to 6 were classified as nonmetropolitan (rural [<250 000 inhabitants]).^15^

We tested for linear trends in the density of primary care clinicians (primary care physicians, nurse practitioners, and physician assistants) using the Jonckheere-Terpstra test for rural and urban counties. A nonparametric method (ie, the Jonckheere-Terpstra test) was used because the outcome measure (density of primary care clinicians) was not normally distributed over the study period. In addition, we presented the descriptive statistics of the mean densities of the 3 types of clinicians from 2009 to 2017 across rural and urban counties and calculated the average annual percentage change (APC) in the means.

To estimate the changes in annual rates of the densities of each type of primary care clinicians by rural and urban counties, we fitted generalized estimating equations adjusting for county-level sociodemographic variables. From the generalized estimating equations analysis, we compared the changes in the densities of primary care clinicians between rural and urban counties over time using the Wald χ^2^ test. County-level sociodemographic variables included the proportion of women, White individuals, Black individuals, Asian individuals, population aged 65 years and over, born outside the US, and population with a 4-year college degree residing in the county. The yearly county-level median household income was categorized according to the published household income quintiles from the Census Bureau Historical Income Tables and was included in the generalized estimating equations analysis.^25^ A 2-tailed *P* < .05 was regarded as statistically significant. Data management and analysis were performed with R, version 3.6.2 (R Foundation for Statistical Computing), and SAS, version 9.4 (SAS Institute Inc).

## Results

**Figure 1.**
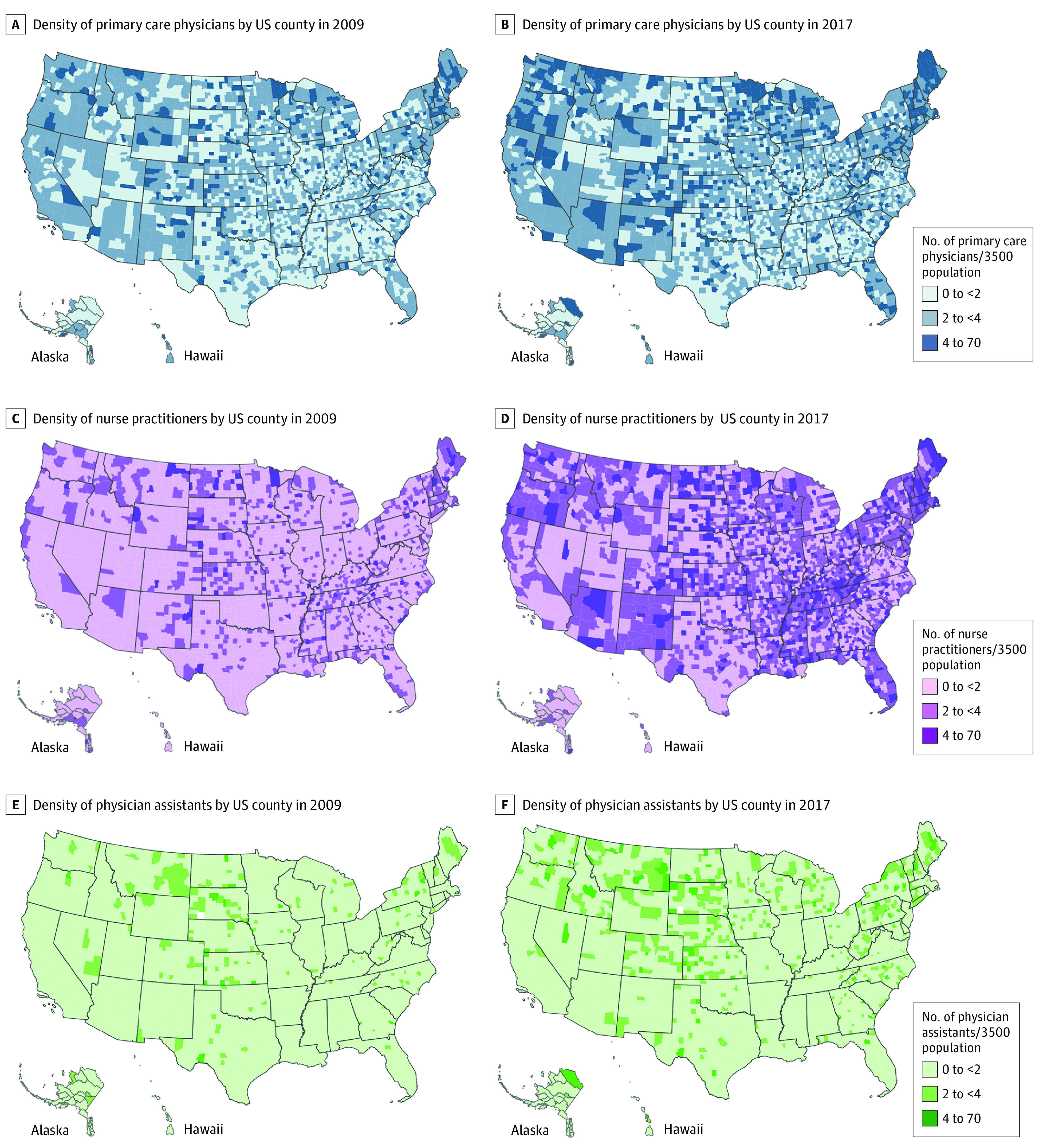
Distribution of Primary Care Physicians and Nonphysician Clinicians Across US Counties, 2009 vs 2017 The map contours conform to the default setting of the mapping platform ArcGIS (esri).

The study included yearly data from 3143 US counties (1167 urban and 1976 rural counties). The trend analysis comparing the distribution of primary care clinicians per 3500 people in 2009 and 2017 reported in Figure 1 depicts a geographic distribution in the density of all 3 types of primary care clinicians across the US. The overall density of primary care clinicians has been increasing from 2009 to 2017 (ie, the 2017 maps in the right panel have higher numbers of darker shade counties than the 2009 maps in the left panel).

**Figure 2.**
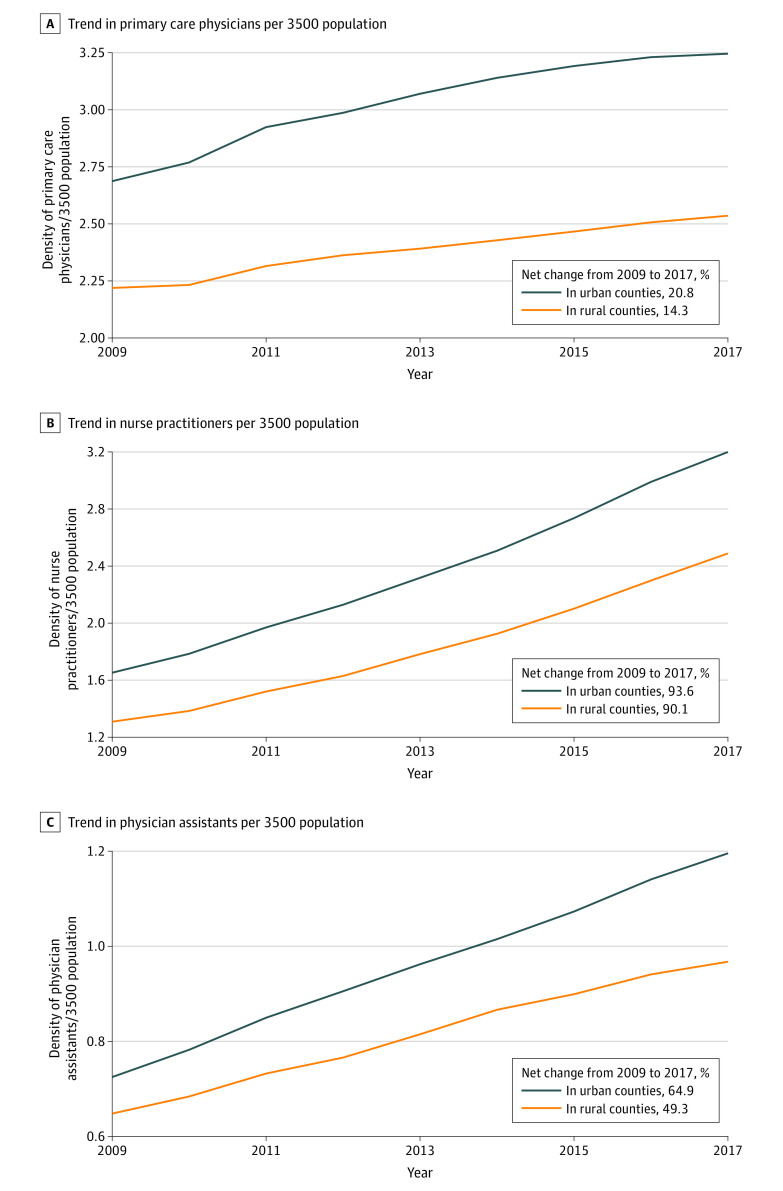
Trends in the Density of Primary Care Clinicians in US Rural vs Urban Counties From 2009 to 2017

Figure 2 shows the trend in the average densities for primary care clinicians in the period from 2009 to 2017. The density of primary care physicians in urban counties increased by 20.8%, from 2.69 in 2009 to 3.25 in 2017. In rural counties, the density increased by 14.3%, from 2.22 in 2009 to 2.54 in 2017. Likewise, the density of nurse practitioners in urban counties increased by 93.6% from 1.65 to 3.20, while in rural counties, it increased by 90.1% from 1.31 to 2.49. The density of physician assistants in urban counties increased by 64.9% from 0.73 to 1.20, and it increased by 49.3% from 0.65 to 0.97 in rural counties.

Table 1 reports the trend analysis and results from the Jonckheere-Terpstra test. The density of primary care physicians increased substantially from 2009 to 2017 in rural counties (2.04 [interquartile range {IQR}, 1.43-2.76] for 2009 vs 2.29 [IQR, 1.57-3.23] for 2017; *P* < .001), for nurse practitioners (1.16 [IQR, 0.61-1.80] for 2009 vs 2.24 [IQR, 1.39-3.30] for 2017; *P* < .001), and for physician assistants (0.43 [IQR, 0.00-0.96] for 2009 vs 0.69 [IQR, 0.16-1.37] for 2017; *P* < .001). Similar increasing trends were observed in urban counties for primary care physicians (2.26 [IQR, 1.52-3.23] for 2009 vs 2.66 [IQR, 1.72-4.02] for 2017; *P* < .001), nurse practitioners (1.28 [IQR, 0.72-2.11] for 2009 vs 2.52 [IQR, 1.61-3.99] for 2017; *P* < .001), and physician assistants (0.56 [IQR, 0.22-0.95] for 2009 vs 0.90 [IQR, 0.42-1.55] for 2017; *P* < .001).

**Table 1.** Medians of the Density of the Different Primary Care Clinicians Stratified by County Rural and Urban Status, 2009-2017

Variable	Clinicians per 3500 individuals, median (interquartile range) No.^a^									*P* for trend^b^
	2009	2010	2011	2012	2013	2014	2015	2016	2017	
**Primary care physicians**										
Urban	2.26 (1.52-3.23)	2.31 (1 .53-3.32)	2.39 (1.61-3.52)	2.46 (1.61-3.60)	2.47 (1.64-3.73)	2.55 (1.65-3.81)	2.57 (1.68-3,88)	2.61 (1.70-3.98)	2.66 (1.72-4.02)	<.001
Rural	2.04 (1.43-2.76)	2.06 (1.42-2.80)	2.13 (1.47-2.89)	2.15 (1.50-2.98)	2.18 (1.52-3.03)	2.21 (1.53-3.08)	2.25 (1.56-3.14)	2.27 (1.56-3.21)	2.29 (1.57-3.23)	<.001
**Nurse practitioners**										
Urban	1.28 (0.72-2.11)	1.37 (0.82-2.27)	1.52 (0.90-2.49)	1.64 (0.98-2.67)	1.82 (1.10-2.88)	1.98 (1.21-3.11)	2.14 (1.36-3.37)	2.35 (1.50-3.69)	2.52 (1.61-3.99)	<.001
Rural	1.16 (0.61-1.80)	1.21 (0.65-1.91)	1.34 (0.72-2.08)	1.41 (0.80-2.23)	1.55 (0.91-2.41)	1.68 (1.01-2.61)	1.88 (1.14-2.83)	2.05 (1.25-3.09)	2.24 (1.39-3.30)	<.001
**Physician assistants**										
Urban	0.56 (0.22-0.95)	0.60 (0.24-1.03)	0.65 (0.27-1.10)	0.70 (0.29-1.17)	0.74 (0.32-1.23)	0.78 (0.35-1.31)	0.82 (0.37-1.39)	0.86 (0.41-1.49)	0.90 (0.42-1.55)	<.001
Rural	0.43 (0.00-0.96)	0.46 (0.00-1.00)	0.49 (0.00-1.06)	0.52 (0.00-1.09)	0.56 (0.10-1.13)	0.61 (0.13-1.21)	0.64 (0.14-1.26)	0.66 (0.15-1.34)	0.69 (0.16-1.37)	<.001

^a^ Median of primary care clinicians over time.

^b^ *P* value for linear trend over the years determined by the Jonckheere-Terpstra test.

Table 2 presents the mean densities of primary care clinicians and the APCs from 2009 to 2017. In urban counties, the APC in the mean density of primary care physicians was 2.40% (95% CI, 1.19%-3.61%), for nurse practitioners was 8.64% (95% CI, 7.72%-9.55%), and for physician assistants was 6.42% (95% CI, 5.34%-7.50%). In rural counties, the APC for the density of primary care physicians was 1.70% (95% CI, 0.84%-2.57%), for nurse practitioners was 8.37% (95% CI, 7.11%-9.63%), and for physician assistants was 5.14% (95% CI, 3.91%-6.37%). The APC in the mean density of primary care clinicians is more prominent in urban than in rural counties.

**Table 2.** Annual Mean Changes in the Density of the Different Primary Care Clinicians in US Rural and Urban Counties, 2009-2017

Variable	Annual mean change of the density of primary care clinicians per 3500 individuals, %								Mean (95% CI)^a^
	2009-2010	2010-2011	2011-2012	2012-2013	2013-2014	2014-2015	2015-2016	2016-2017	
**Primary care physician**									
Urban	2.97	5.42	2.40	2.68	2.28	1.59	1.25	0.62	2.40 (1.19-3.61)
Rural	0.45	4.04	1.72	1.27	1.67	1.65	1.62	1.20	1.70 (0.84-2.57)
**Nurse practitioner**									
Urban	7.88	10.67	8.12	8.92	8.19	9.16	9.12	7.02	8.64 (7.72-9.55)
Rural	5.34	10.14	7.24	9.20	8.43	8.81	9.52	8.26	8.37 (7.11-9.63)
**Physican assistant**									
Urban	6.85	8.97	7.06	5.49	6.25	4.90	6.54	5.26	6.42 (5.34-7.50)
Rural	4.62	7.35	5.48	6.49	6.10	3.45	4.44	3.19	5.14 (3.91-6.37)

^a^ Calculated by averaging the annual mean changes of the density of primary care clinicians over time.

Results from the generalized estimating equations analysis adjusting for county-level sociodemographic characteristics are shown in Table 3. The adjusted estimates show that the densities of primary care physicians (β = 0.04; 95% CI, 0.03-0.05; *P* < .001), nurse practitioners (β = 0.16; 95% CI, 0.15-0.16; *P* < .001), and physician assistants (β = 0.04; 95% CI, 0.04-0.05; *P* < .001) have been increasing during the study period, although at faster rates in urban counties. The difference in annual changes of the primary care physician density between urban and rural counties was significant (β = 0.03; 95% CI, 0.02 to 0.04; *P* < .001). Similarly, the density of nurse practitioners was also increasing faster in urban counties than in rural counties (β = 0.05; 95% CI, 0.03-0.06; *P* < .001). The same discrepancy between urban and rural areas was observed in changes of the physician assistant density (β = 0.02; 95% CI, 0.01-0.02; *P* < .001). The density of primary care physicians was positively associated with the median household income categories, the proportion of Asian residents (β = 7.04; 95% CI, 2.37-11.71; *P* = .003), and residents with a college degree (β = 1.37; 95% CI, 0.04-2.69; *P* = .04). But there was no significant association between the median household income quintiles and the density of nurse practitioners. Counties with a higher proportion of Black (β = 1.01; 95% CI, 0.33-1.70; *P* = .004) and Asian residents (β = 4.45; 95% CI, 1.46-7.44; *P* = .004) were more likely to have a higher density of nurse practitioners. In contrast, an inverse association was observed between the proportion of Black individuals (β = −1.24; 95% CI, –1.63 to –0.85 *P* < .001) and the density of physician assistants.

**Table 3.** Association Between Density of Primary Care Clinicians and Sociodemographic Characteristics in US Rural and Urban Counties, 2009-2017^a^

Parameters	Primary care physicians		Nurse practitioners		Physician assistants	
	β (95% CI)	*P* value	β (95% CI)	*P* value	β (95% CI)	*P* value
Intercept	1.41 (0.63 to 2.18)	<.001	1.25 (0.26 to 2.24)	.01	1.15 (0.53 to 1.78)	<.001
Rural^b^	NA	NA	NA	NA	NA	NA
Urban	0.39 (0.20 to 0.57)	<.001	0.16 (0.04 to 0.28)	.01	0.03 (–0.05 to 0.10)	.51
Year	0.04 (0.03 to 0.05)	<.001	0.16 (0.15 to 0.16)	<.001	0.04 (0.04 to 0.05)	<.001
Year × urban	0.03 (0.02 to 0.04)	<.001	0.05 (0.03 to 0.06)	<.001	0.02 (0.01 to 0.02)	<.001
Median household income, quintile						
Lowest (<1st)	NA	NA	NA	NA	NA	NA
1st to 2nd, inclusive	0.15 (0.05 to 0.26)	.004	–0.18 (–0.54 to 0.17)	.3	0.10 (0.02 to 0.18)	.02
2nd to 3rd, inclusive	0.14 (0.03 to 0.25)	.01	–0.27 (–0.62 to 0.08)	.13	0.10 (0.01 to 0.19)	.03
≤3rd to 4th, inclusive	0.14 (0.01 to 0.27)	.03	–0.24 (–0.61 to 0.14)	.22	0.09 (–0.01 to 0.18)	.07
Highest (>4th)	0.04 (–0.26 to 0.35)	.78	–0.29 (–0.76 to 0.19)	.24	–0.01 (–0.22 to 0.20)	.91
Woman, %	0.56 (–0.72 to 1.84)	.39	0.90 (–0.58 to 2.38)	.23	0.06 (–0.95 to 1.08)	.90
Age ≥65 y, %	–0.12 (–1.35 to 1.10)	.84	–2.84 (–4.40 to – 1.28)	<.001	–0.75 (–1.52 to 0.01)	.05
White, %	0.30 (–0.33 to 0.93)	.35	0.09 (–0.46 to 0.64)	.75	–0.54 (–0.90 to – 0.17)	.004
Black, %	0.64 (–0.14 to 1.42)	.11	1.01 (0.33 to 1.70)	.004	–1.24 (–1.63 to – 0.85)	<.001
Asian, %	7.04 (2.37 to 11.71)	.003	4.45 (1.46 to 7.44)	.004	2.85 (1.00 to 4.70)	.003
Born outside the US, %	1.69 (–3.15 to 6.53)	.49	3.14 (–4.93 to 11.21)	.45	0.37 (–3.17 to 3.90)	.84
With college education, %	1.37 (0.04 to 2.69)	.04	1.10 (–0.27 to 2.47)	.12	0.54 (–0.37 to 1.45)	.24

Abbreviation: NA, not applicable.

^a^ Estimates from the generalized estimating equations.

^b^ National Center for Health Statistics 2013 Urban-Rural Classification Scheme for Counties.^15^

## Discussion

This cross-sectional study examined trends in the density of primary care physicians, nurse practitioners, and physician assistants across rural and urban counties in the US from 2009 to 2017. Overall, there were increasing trends in the density of each type of primary care clinicians across all counties. While there is an overall increase, our study revealed significant geographic disparities in the density of primary care clinicians (primary care physicians, nurse practitioners, and physician assistants) between rural and urban areas. Rural-urban disparities widened during the study period. Moreover, the rural-urban differences continued to be substantial and significant after adjusting for county-level sociodemographic variables.

Prevalence of chronic disease is 1 of the major contributors to rising health care costs in the US. For rural residents who are at a higher risk of developing coronary heart disease and stroke, greater access to primary care clinicians would improve the coordination of care and health outcomes.^2,3,26^ Furthermore, the shortage of primary care clinicians may be related to worse health outcomes in US rural counties.^7,9,27^ Basu et al^27^ found an association between primary care physician supply and mortality in the US. These outcomes have been analyzed with socioeconomic status, health care coverage, and travel distance to health care facilities, in addition to access to primary care clinicians.^13,27,28,29,30,31,32^

To our knowledge, this study is the first to analyze trends in density of primary care clinicians in rural and urban counties adjusting for sociodemographic factors that may be associated with the characteristics of each county. We found a positive linear correlation between the number of primary care clinicians per 3500 people and median household income quintile categories, and the proportion of some race/ethnicity categories.

The findings have implications for policy. For example, programs and policies to improve access to and quality of care in rural areas such as geographically based adjustments in Medicare payment,^33^ or programs that encourage clinical practice in rural areas may be necessary to reduce the gaps. State actions related to the expansion of Medicaid have led to hospital closures in rural areas,^34^ which might act as a disincentive for physicians to practice in rural areas. The extensive availability of Medicaid coverage provides the needed linkages and payment infrastructure for primary care clinicians to serve patients in need of care but with limited resources.^35^ In addition, studies have shown that primary care provided by nonphysician clinicians such as nurse practitioners and physician assistants are increasingly accepted by patients.^36^ However, variation in practice regulations across states and communities exists, and some regions may lag behind in the national trend of growing nonphysician clinicians.^37^ Furthermore, the expansion of telemedicine has the potential to improve access, use, and outcomes among rural residents, partially mitigating health disparities due to rural clinician shortages.^38,39^

### Limitations

This study has 3 key limitations. First, because it is difficult to dissect the complex employment-related decision-making process of individual clinicians, including dual careers, childcare, cultural background and geographic attachment, our analysis is not immune to omitted variable biases. Second, many factors may affect the supply of primary care clinicians. The number of explanatory variables for a county in this study was limited by the availability of existing data sources and, thus, our conclusions may be difficult to generalize. For example, international medical graduates contribute to rural health in some states by helping reduce rural health care workforce gaps.^40,41^ The National Plan and Provider Enumeration System does not contain individual-level information such as age, gender, race/ethnicity and place of birth, thus, we were unable to assess the association between clinicians born outside the US and the size and change over time of the primary care workforce in rural and urban counties, particularly with regard to health professional shortage areas. Third, we did not use a causal approach to assessing the factors contributing to changes over time of the primary care workforce in our generalized estimating equations analysis given data limitations.

## Conclusions

While the density of primary care clinicians increased in both rural and urban counties, the pace of growth of primary care clinicians in urban counties has been faster than that in rural counties from 2009 to 2017. Reducing urban-rural disparities in access to primary care clinicians requires policy and intervention efforts that can meaningfully increase the supply of primary care clinicians in rural areas.

## References

[1] Meit M, Knudson A, Gilbert T The 2014 update of the rural-urban chartbook. Accessed September 30, 2020. https://ruralhealth.und.edu/projects/health-reform-policy-research-center/pdf/2014-rural-urban-chartbook-update.pdf

[2] Leira EC, Hess DC, Torner JC, Adams HP Jr Rural-urban differences in acute stroke management practices: a modifiable disparity. Arch Neurol. 2008;65(7):887-891. doi:10.1001/archneur.65.7.887 18625855

[3] O’Connor A, Wellenius G Rural-urban disparities in the prevalence of diabetes and coronary heart disease. Public Health. 2012;126(10):813-820. doi:10.1016/j.puhe.2012.05.029 22922043

[4] Kulshreshtha A, Goyal A, Dabhadkar K, Veledar E, Vaccarino V Urban-rural differences in coronary heart disease mortality in the United States: 1999-2009. Public Health Rep. 2014;129(1):19-29. doi:10.1177/003335491412900105 24381356 PMC3863000

[5] Singh GK, Siahpush M Widening rural-urban disparities in life expectancy, U.S., 1969-2009. Am J Prev Med. 2014;46(2):e19-e29. doi:10.1016/j.amepre.2013.10.017 24439358

[6] Starfield B, Shi L, Macinko J Contribution of primary care to health systems and health. Milbank Q. 2005;83(3):457-502. doi:10.1111/j.1468-0009.2005.00409.x 16202000 PMC2690145

[7] Ricketts TC Workforce issues in rural areas: a focus on policy equity. Am J Public Health. 2005;95(1):42-48. doi:10.2105/AJPH.2004.047597 15623856 PMC1449848

[8] Kirby JB, Yabroff KR Rural-urban differences in access to primary care: beyond the usual source of care provider. Am J Prev Med. 2020;58(1):89-96. doi:10.1016/j.amepre.2019.08.026 31862103

[9] MacDowell M, Glasser M, Fitts M, Nielsen K, Hunsaker M A national view of rural health workforce issues in the USA. Rural Remote Health. 2010;10(3):1531. 20658893 PMC3760483

[10] Rosenblatt RA, Hart LG Physicians and rural America. West J Med. 2000;173(5):348-351. doi:10.1136/ewjm.173.5.348 11069878 PMC1071163

[11] Bowman RC Measuring primary care: the standard primary care year. Rural Remote Health. 2008;8(3):1009. 18785798

[12] Friedberg MW, Hussey PS, Schneider EC Primary care: a critical review of the evidence on quality and costs of health care. Health Aff (Millwood). 2010;29(5):766-772. doi:10.1377/hlthaff.2010.0025 20439859

[13] Hawks L, Himmelstein DU, Woolhandler S, Bor DH, Gaffney A, McCormick D Trends in unmet need for physician and preventive services in the United States, 1998-2017. JAMA Intern Med. 2020;180(3):439-448. doi:10.1001/jamainternmed.2019.6538 31985751 PMC6990729

[14] Miller ME, Zuckerman S Comparing urban and rural physicians. Health Aff (Millwood). 1991;10(4):243-253. doi:10.1377/hlthaff.10.4.243 1778559

[15] Ingram DD, Franco SJ NCHS urban-rural classification scheme for counties. Vital Health Stat 2. 2012;(154):1-65. 22783637

[16] Hall SA, Kaufman JS, Ricketts TC Defining urban and rural areas in U.S. epidemiologic studies. J Urban Health. 2006;83(2):162-175. doi:10.1007/s11524-005-9016-3 16736366 PMC2527174

[17] National Academies of Sciences, Engineering, and Medicine; Health and Medicine Division; Board on Population Health and Public Health Practice; Roundtable on the Promotion of Health Equity; Roundtable on Population Health Improvement Achieving Rural Health Equity and Well-Being: Proceedings of a Workshop. National Academies Press; 2018. 30307731

[18] Billi JE, Pai CW, Spahlinger DA The effect of distance to primary care physician on health care utilization and disease burden. Health Care Manage Rev. 2007;32(1):22-29. doi:10.1097/00004010-200701000-00004 17245199

[19] Brundisini F, Giacomini M, DeJean D, Vanstone M, Winsor S, Smith A Chronic disease patients’ experiences with accessing health care in rural and remote areas: a systematic review and qualitative meta-synthesis. Ont Health Technol Assess Ser. 2013;13(15):1-33. 24228078 PMC3817950

[20] Buykx P, Humphreys J, Wakerman J, Pashen D Systematic review of effective retention incentives for health workers in rural and remote areas: towards evidence-based policy. Aust J Rural Health. 2010;18(3):102-109. doi:10.1111/j.1440-1584.2010.01139.x 20579020

[21] National Provider Identifier record. Accessed September 30, 2020. https://www.cms.gov/Regulations-and-Guidance/Administrative-Simplification/NationalProvIdentStand/DataDissemination

[22] US Census Bureau Accessed September 30, 2020. https://www.census.gov/programs-surveys/acs/data.html

[23] von Elm E, Altman DG, Egger M, Pocock SJ, Gøtzsche PC, Vandenbroucke JP; STROBE Initiative The Strengthening the Reporting of Observational Studies in Epidemiology (STROBE) statement: guidelines for reporting observational studies. J Clin Epidemiol. 2008;61(4):344-349. doi:10.1016/j.jclinepi.2007.11.008 18313558

[24] Kaiser Family Foundation Primary care health professional shortage areas. Accessed September 30, 2020. https://www.kff.org/other/state-indicator/primary-care-health-professional-shortage-areas-hpsas/?currentTimeframe=0&sortModel=%7B%22colId%22:%22Location%22,%22sort%22:%22asc%22%7D

[25] US Census Bureau Historical Income Tables Tables H1 and H3 2019 Accessed September 30, 2020. https://www.census.gov/data/tables/time-series/demo/income-poverty/historical-income-households.html

[26] Clark RA, Eckert KA, Stewart S, Rural and urban differentials in primary care management of chronic heart failure: new data from the CASE study. Med J Aust. 2007;186(9):441-445. doi:10.5694/j.1326-5377.2007.tb00993.x 17484704

[27] Basu S, Berkowitz SA, Phillips RL, Bitton A, Landon BE, Phillips RS Association of primary care physician supply with population mortality in the United States, 2005-2015. JAMA Intern Med. 2019;179(4):506-514. doi:10.1001/jamainternmed.2018.7624 30776056 PMC6450307

[28] Aboagye JK, Kaiser HE, Hayanga AJ Rural-urban differences in access to specialist providers of colorectal cancer care in the United States: a physician workforce issue. JAMA Surg. 2014;149(6):537-543. doi:10.1001/jamasurg.2013.5062 24740165

[29] Hao Y, Landrine H, Jemal A, Race, neighbourhood characteristics and disparities in chemotherapy for colorectal cancer. J Epidemiol Community Health. 2011;65(3):211-217. doi:10.1136/jech.2009.096008 19959651

[30] Roetzheim RG, Pal N, Gonzalez EC, Ferrante JM, Van Durme DJ, Krischer JP Effects of health insurance and race on colorectal cancer treatments and outcomes. Am J Public Health. 2000;90(11):1746-1754. doi:10.2105/AJPH.90.11.1746 11076244 PMC1446414

[31] Xue Y, Smith JA, Spetz J Primary care nurse practitioners and physicians in low-income and rural areas, 2010-2016. JAMA. 2019;321(1):102-105. doi:10.1001/jama.2018.17944 30620363 PMC6583766

[32] Odisho AY, Fradet V, Cooperberg MR, Ahmad AE, Carroll PR Geographic distribution of urologists throughout the United States using a county level approach. J Urol. 2009;181(2):760-765. doi:10.1016/j.juro.2008.10.034 19091334

[33] Steinwald AB, Sloan FA, Edmunds M Geographic Adjustment in Medicare Payment: Phase II: Implications for Access, Quality, and Efficiency. National Academies Press; 2012. 24921116

[34] Hoadley J, Alker J, Homes M Health insurance coverage in small towns and rural America: the role of Medicaid expansion. Accessed September 30, 2020. https://ccf.georgetown.edu/wp-content/uploads/2018/09/FINALHealthInsuranceCoverage_Rural_2018.pdf

[35] Rajbhandari-Thapa J, Zhang D, MacLeod KE, Thapa K Impact of Medicaid expansion on insurance coverage rates among adult populations with low income and by obesity status. Obesity (Silver Spring). 2020;28(7):1219-1223. doi:10.1002/oby.22793 32304356 PMC8627371

[36] Dill MJ, Pankow S, Erikson C, Shipman S Survey shows consumers open to a greater role for physician assistants and nurse practitioners. Health Aff (Millwood). 2013;32(6):1135-1142. doi:10.1377/hlthaff.2012.1150 23733989

[37] American Medical Association Physician assistant scope of practice. Accessed May 1, 2018. https://www.ama-assn.org/sites/ama-assn.org/files/corp/media-browser/public/arc-public/state-law-physician-assistant-scope-practice.pdf

[38] Kruse CS, Bouffard S, Dougherty M, Parro JS Telemedicine use in rural Native American communities in the era of the ACA: a systematic literature review. J Med Syst. 2016;40(6):145. doi:10.1007/s10916-016-0503-8 27118011 PMC4848328

[39] Zhang D, Wang G, Zhu W, Expansion of telestroke services improves quality of care provided in super rural areas. Health Aff (Millwood). 2018;37(12):2005-2013. doi:10.1377/hlthaff.2018.05089 30633675

[40] Thompson MJ, Hagopian A, Fordyce M, Hart LG Do international medical graduates (IMGs) “fill the gap” in rural primary care in the United States? a national study. J Rural Health. 2009;25(2):124-134. doi:10.1111/j.1748-0361.2009.00208.x 19785577

[41] Boulet JR, Duvivier RJ, Pinsky WW Prevalence of international medical graduates from Muslim-majority nations in the US physician workforce from 2009 to 2019. JAMA Netw Open. 2020;3(7):e209418. doi:10.1001/jamanetworkopen.2020.9418 32663311 PMC7339131

